# Supplementary material for: Metric information in cognitive maps: Euclidean embedding of non-Euclidean environments
Source: PLoS Comput Biol. 2023 Dec 27;19(12):e1011748. doi: 10.1371/journal.pcbi.1011748 (PMC10775987; doi:10.1371/journal.pcbi.1011748)
Supplement: Attachment — Submitted filename: rebuttal.pdf [file pcbi.1011748.s001.pdf]

Tristan Baumann
Computational Neuroscience
Max Planck Institute for Biological Cybernetics
Max-Planck-Ring 8
72076 Tübingen, Germany
tristan.baumann@tuebingen.mpg.de
tristan.baumann@student.uni-tuebingen.de

Dear Editors,

Please find below our point-by-point reply to the reviewers' remarks. The comments and questions of the reviewers are shown in blue, followed by our reply in black. Modifications to the manuscript are shown in red. The replies also cover your recommendation:

In particular, the authors should make use of Bayesian statistics or information criteria for model selection (e.g. AIC or BIC) to make a more formal comparison between their embedding model and a topological framework; and - if possible - attempt to relate their embedding model to a wider range of published data sets, to ensure that their conclusions are robust.

Best regards,
Tristan Baumann (on behalf of the co-author)

Reviewer #1: Summary

In this paper the authors propose a model that can account for deviation from Euclidean space in cognitive maps holding on the hypothesis that cognitive maps are fundamentally Euclidean. Basically, they suggest that, instead of learning and representing the position of objects/events in space using a non-euclidean graphical structure, what people usually do is to embed a non-metric model in 2D Euclidean coordinates. By re-analyzing the data from a previous behavioral experiment, they show that the embedded model and the graph model (non Euclidean) explain the data equally well. Although the embedded model, thus, is not necessarily better than a graph model, the former is preferable because it allows to integrate local repeated measures into a global structure (whereas in a non-metric graph-like cognitive map, each edge is independent from the others).

I liked the paper, it investigates a very important as well as difficult question: Whether humans navigate the environment constructing an Euclidean map. The author suggest that this is the case, proposing a hybrid model in which a non-Euclidean graph is embedded in a 2d Euclidean coordinates and re-analyzing an important dataset that, putatively, provide evidence for non-Euclidean navigation. I find the paper well written and, to the level of understanding that I can provide, which does not cover all the mathematical details as well as all the relevant literature on this specific argument (cognitive maps vs cognitive graphs), it seems solid: I could not find any specific problem in the analysis or the construction of the model.

Although, at the end, the paper cannot adjudicate between the labeled graph and the embedded graph model, I think it will be a good addition to the literature and the current debate.

We thank the reviewer for their excellent review and the important concerns raised. We have made significant changes and additions to the manuscript, as detailed in the following:

Concerns raised:

1. The author can report Bayesian statistics to support the lack of difference between models.

This is a great suggestion. A similar concern was also raised by reviewer #2, who proposed the Bayesian information criterion (BIC) as a measure. We used the BIC to compare the two models and found that it lends strong support to the Euclidean metric embedding. For the modifications to the manuscript and further comments, please refer to Review #2.1.

2. Is there another dataset on which the two models can be compared? It would be more convincing to see a replication of the results using different data (with different non-Euclidean environments).

While there have been numerous studies over the years that found large non-Euclidean biases in judgments about distance, position, or orientation (e.g., Tversky (1992); McNamara and Diwadkar (1997)), active navigation through non-Euclidean environments is relatively rare. One such study would be Ruddle et al. (2000), in which participants navigated a series of virtual rooms connected either directly by doors or via wormholes that bridged large distances. While the setup is reminiscent of Warren et al. (2017), Ruddle et al. (2000) did not report any pose data or measures of distances and angles in their virtual maze. It might be possible to fit our model to sketch maps from the (Ruddle et al., 2000) study, but this would require some heavy assumptions about the relation between the sketches and the actual mental map. We are not aware of any studies other than Warren et al. (2017) that investigate non-Euclidean environments with walking subjects.

3. P. 16 "The embedding may possibly be somewhat better at predicting the within-subject angular deviation in the rips and folds dataset, but the results did not pass the selected significance threshold" – I suggest to delete this sentence from the manuscript. The difference is not significant and it should not be commented as a hint of a possible difference.

> **Modification #1, Removed sentence on p. 16 (4 Discussion)**

[...] with a similar magnitude and distribution. ~~The embedding may possibly be somewhat better at predicting the within-subject angular deviation in the rips and folds dataset, but the results did not pass the selected significance threshold at $\alpha = .05$.~~ Given the data [...]

Review #2: Summary

The study utilized data from Warren et al. (2017) to investigate whether embedding a graph into Euclidean coordinates can explain data from a wormhole experiment better than a labeled graph. They used a numerical optimization method to derive embeddings, which are representations of the cognitive map. For their primary dataset, they derived shortcut predictions from both the non-metric labeled and embedded graph models. These predictions were then compared to human shortcut estimates from the Warren et al. (2017) study. The authors found that both the Euclidean embedding and the non-metric model predicted the data with similar accuracy. They conclude that since the embedding graph is simpler, it is a better model for the cognitive map than the non-metric models.
The authors employed a unique approach by using a numerical optimization method to derive embeddings, and the use of both non-metric labeled and embedded graph models to predict human behavior in navigating non-Euclidean environments is a solid approach. This approach takes the question about non-metric graphs into a new phase of direct comparisons between models. Given that most of the data were derived from another paper, design questions are minimal.

We thank the reviewer for their excellent review and the important concerns raised. We have made significant changes and additions to the manuscript, as detailed in the following:

Major concerns:

1. The biggest concern is about the model comparison. The two models predicted the data with similar accuracy, which could lead to ambiguity in comparing the two models. The authors assume that the Euclidean embedded model is simpler, but there is not really much to support that claim. Conducing AIC or BIC to more directly compare the models would help, or at least providing more justification as to why metric embedding is simpler. A topological graph seems like a fairly simple idea to me. The metric embedding yields multi-level paths (e.g., Figures 4 and 5), which seems like it could be far more complex than the topological graph, and is also non-Euclidean.

The addition of the BIC for model comparison is an excellent suggestion and actually strongly favors our model: Leveraging metric constraints, the Euclidean embedding needs much fewer parameters to be fully specified compared to the non-metric labeled graph. This difference in parameters leads to a lower ore for the embedding. We added the following passages to the manuscript:

> Modification #2, p. 13 (2.3 Model comparison and data analysis)

> We then compared the models using the Bayesian information criterion (BIC, Schwarz (1978)). The BIC is based on the maximum likelihood of observing the data given a specific model and penalizes the number of free parameters in the model. Generally, a model with a lower BIC is preferable.
>
> To obtain likelihood functions for the prediction errors, we added a noise term describing the inter-subject variation to both models. The noise was modeled as a von Mises distribution using the empirical prediction error means and variances. In the embedded graph, the free parameters are the $n \times 2$ coordinates of the 2D Euclidean embedding X and a noise term for a total of 82 free parameters. To fully specify the non-metric labeled graph model, the required parameters are the set of all distance labels d and angle labels α, as well as the noise term for a total of 162 parameters. Note that these definitions are only valid in the respective Euclidean- and graph-based frameworks which, for example, come with different distance functions (straight lines in the Euclidean framework and the shortest path in the graph-based framework).

> Modification #3, p. 16 (3.2 Dataset 1: Route-finding and shortcuts)

Given the similar prediction errors but large difference in free parameters, the Bayesian information criterion strongly favored the embedding over the non-metric labeled graph ($\text{BIC}_{\text{embedding}} = 221.7$ vs. $\text{BIC}_{\text{non-metric}} = 405.97$).

> **Modification #4, p. 16 (3.3 Dataset 2: Rips and folds)**
The Bayesian information criterion also again strongly favored the Euclidean embedding over the non-metric alternative ($\text{BIC}_{\text{embedding}} = 233.66$ vs. $\text{BIC}_{\text{non-metric}} = 425.92$).

> **Modification #5, p. 17 (4 Discussion)**
The Bayesian information criterion, on the other hand, strongly favored the Euclidean embedding in both cases. Due to the similar predictions, the difference in BIC scores are largely a result of the differences in free parameters needed to fully specify the models. In this sense, the metric constraints of the Euclidean embedding are advantageous, leading to a simpler model. In the non-metric labeled graph, each label is independent of other labels and must therefore be fully specified. Still, due to the non-Euclidean property of the wormhole environment, a perfect Euclidean embedding cannot exist and a difference between the models must remain. It is therefore surprising that the lack of metric constraints in the non-metric labeled graph did not lead to significantly different prediction errors.

> **Modification #6, p. 17-18 (4 Discussion)**
This finding is also strongly supported by the large difference in BIC score between the models arising from the difference in required parameters to specify them. While the less-constrained mental map mental map may seem advantageous to account for navigational deviations from Euclidean metrics, in the present study, it was not.

We also improved the description of Figure 4d to illustrate how the intersecting edges of the embedding could be realized in a mental map:

> **Modification #7, p.14 (Caption Fig. 4d)**
(d) Sketch of the distorted wormhole maze according to the embedding in (c). The sketch shows how the embedding might be represented by a subject. Edges that cross each other in the embedded graph could for example be rationalized as multi-level paths, leading to a 3D representation. Alternatively, in a purely 2D map, the arms would simply intersect. Note that the edges have no coordinates in the embedding but are simply lines in the adjacency matrix.

2. Is the stress function simpler than a set of heuristics, such as regularizing to 90 degrees?

Our algorithm is unbiased in that it successfully finds an embedding that minimizes the differences between vertex positions and graph labels. It might be possible to capture a 90 degree bias via the labels of the graph (e.g., by rounding angles close to 90 degrees), but this would at best be a fixed term and therefore not change the complexity of the models. Given the many oblique angles of the wormhole maze, we believe it is unlikely that simplifying the stress function to multiples of 90 degrees would lead to a better fit of the maze, if it found any solution at all. We are not aware of other embedding stress functions without the requirement of a global reference direction.

3. How does the embedding explain inconsistencies in regular Euclidean judgments, e.g., Diwadkar & McNamara, Tversky 1992, etc.? If the metric embedding simplifies to Euclidean structure in normal environments (see comment below), then how can it explain other violations of Euclidean geometry observed in the literature?

The argument for the present data is basically that the observed deviations from the Euclidean ground truth arise from a skewed representation, which may however still be Euclidean. It is true that a purely Euclidean representation can never account for asymmetric distance judgments like the ones reported in McNamara and Diwadkar (1997) or Tversky (1992). However, there is a difference between the form of a representation and the inferences drawn from that representation, which leaves room for e.g. biased distance judgments. Admittedly, these factors are difficult or perhaps even impossible to disentangle, but the same is also true for the labeled graph model. In this particular case, we argue that the metric embedding and the labeled graphs are models for spatial long-term memory while distance comparisons primarily use the spatial (or visual) working memory. We added these thoughts to the discussion:

> **Modification #8, p. 17-18 (4 Discussion)**

Importantly, an internal Euclidean representation also does not preclude the possibility of biased *inference* about the world from that map. For example, it has long been known that judgments about distance and directions between places are biased by context and asymmetric (Tversky, 1992; McNamara and Diwadkar, 1997), which is in principle incompatible with a Euclidean metric map. However, it is possible to arrive at biased estimations if the estimation function itself is biased and context-dependent, even if the representation is not (McNamara and Diwadkar, 1997). Here, distances and angles are explicitly not stored in the metric Euclidean representation and have to be inferred, which leaves room for such biases. Of course, it is difficult to disentangle factors caused by the representation from factors caused by the processing (Tversky, 1992), but this is also true for judgments derived from the labeled graph model, and there is room for compromise:

Minor concerns:

4. Metric embedding is central to this paper, but I didn't see a really clear definition that could be operationalized (or tested/falsified in the future). It starts to come out on line 202, but an earlier definition would allow the reader to follow the arguments in the introduction. Similarly, details about the non-metric labeled graph model only come out on page 12. Much of those details are in the other paper, but a brief description (e.g., averaging of paths found by vector addition) would be useful.

We added a brief description of the intent behind the term "Metric embedding" to its first mentions:

> **Modification #9, p. 5 (1.1 The cognitive map)**

Overall, distance errors in distorted metric maps might even be smaller than in non-metric labeled graphs, where distance labels are independent. A means to find such distorted metric representations by efficiently exploiting all available distance information, is *metric embedding*.

> **Modification #10, p. 6 (1.2 Distorted maps and non-Euclidean environments)**

This may for example be achieved by *metric embedding* (Fig. 1b). Metric embedding is a means of finding a representation of the non-metric labeled graph in 2D or 3D Euclidean space in a way that best reproduces the spatial information contained in the graph.

5. When the authors say the embedding is not a simple averaging and so it won't end up in the middle, it is not clear how that calculation is made to get the new vector.

The embedding is a result of the minimization of the stress function and takes all distance and angle measurements into account, rather than just the closest local position information.

> **Modification #11, p. 8 (1.3 Evidence from wormhole experiments)**

However, the metric embedding is not a simple integration of local position information (Warren's "inertial coordinates") but the mutual consolidation of distance and angle information over the entire graph. I.e., two different positions in undistorted ground truth coordinates may very well occupy the same position in the distorted embedding and vice-versa. Therefore, deviations from the ground truth do not imply that the representation is not Euclidean, but only that the representation does not match the ground truth.

6. How accurate are the measurements of the distances and angles for each triplet (e.g. lines 265)? If they are all very accurate, wouldn't that lead to a Euclidean structure? Or is that the case if you are dealing with a metric map, but not with a wormhole? I think this might have been brought up later on but was not completely clear to me.

The labels of the topological graph exactly match the ground truth; The only difference between the non-metric graph and the metric embedding are due to the the wormhole, which cannot be correctly represented in a metric Euclidean map. The embedding instead minimizes the difference between the vertex positions and the local labels to account for the distortion caused by the wormhole.

> **Modification #12, p. 10-11 (2.2 Graph and map setup)**

For each triplet, the local distances d_{ij}, d_{jk} and the turning angle α_{ijk} were measured in the ground truth maze and added as labels to the topological graph. d_{ij} and d_{jk} describe the distances between places i, j and j, k and α_{ijk} the heading change at j when moving from i to k. All labels were taken from the required egomotion steps such that labels around wormholes differed from the Euclidean ones. I.e., the labeled graph perfectly matches the local geometry encountered throughout the maze, including the passage through wormholes.

7. Abstract line 20 "so" should probably be "so-called"?

> **Modification #13, p. 1 (Abstract)**

We argue that the embedding naturally arises [...]

Other modifications:

> **Modification #14, Title page:** Updated author address.

> **Modification #15, Figure references:** Fixed inconsistent references to sub-figures which sometimes used uppercase letters.

> **Modification #16, Whole text:** Addressed a few spelling mistakes.
